# RBP4 promotes denervation‐induced muscle atrophy through STRA6‐dependent pathway

**DOI:** 10.1002/jcsm.13518

**Published:** 2024-06-21

**Authors:** Kang‐Zhen Zhang, Jia‐Wen Li, Jin‐Shui Xu, Zheng‐Kai Shen, Yu‐Shuang Lin, Can Zhao, Xiang Lu, Yun‐Feng Rui, Wei Gao

**Affiliations:** ^1^ Department of Geriatrics, Zhongda Hospital, School of Medicine Southeast University No. 87 Dingjiaqiao Nanjing Jiangsu China; ^2^ Jiangsu Province Center for Disease Control and Prevention Nanjing China; ^3^ Department of Geriatrics Sir Run Run Hospital, Nanjing Medical University Nanjing China; ^4^ Department of Orthopaedics, Zhongda Hospital, School of Medicine Southeast University No. 87 Dingjiaqiao Nanjing Jiangsu China

**Keywords:** skeletal muscle atrophy, denervation, fat infiltration, RBP4, STRA6

## Abstract

**Backgrounds:**

Fat infiltration of skeletal muscle has been recognized as a common feature of many degenerative muscle disorders. Retinol binding protein 4 (RBP4) is an adipokine that has been demonstrated to be correlated with the presence and severity of sarcopenia in the elderly. However, the exact role and the underlying mechanism of RBP4 in muscle atrophy remains unclear.

**Methods:**

Denervation‐induced muscle atrophy model was constructed in wild‐type and RBP4 knockout mice. To modify the expression of RBP4, mice were received intramuscular injection of retinol‐free RBP4 (apo‐RBP4), retinol‐bound RBP4 (holo‐RBP4) or oral gavage of RBP4 inhibitor A1120. Holo‐RBP4‐stimulated C2C12 myotubes were treated with siRNAs or specific inhibitors targeting signalling receptor and transporter of retinol 6 (STRA6)/Janus kinase 2 (JAK2)/Signal transducer and activator of transcription 3 (STAT3) pathway. Fat accumulation, myofibre cross‐sectional area, myotube diameter and the expression of muscle atrophy markers and myogenesis markers were analysed.

**Results:**

The expression levels of RBP4 in skeletal muscles were significantly up‐regulated more than 2‐fold from 7 days and sustained for 28 days after denervation. Immunofluorescence analysis indicated that increased RBP4 was localized in the infiltrated fatty region in denervated skeletal muscles. Knockout of RBP4 alleviated denervation‐induced fatty infiltration and muscle atrophy together with decreased expression of atrophy marker Atrogin‐1 and MuRF1 as well as increased expression of myogenesis regulators MyoD and MyoG. By contrast, injection of retinol‐bound holo‐RBP4 aggregated denervation‐induced ectopic fat accumulation and muscle atrophy. Consistently, holo‐RBP4 stimulation also had a dose‐dependent effect on the reduction of C2C12 myotube diameter and myofibre cross‐sectional area, as well as on the increase of Atrogin‐1and MuRF1 expression and decrease of MyoD and MyoG expression. Mechanistically, holo‐RBP4 treatment increased the expression of its membrane receptor STRA6 (>3‐fold) and promoted the phosphorylation of downstream JAK2 and STAT3. Inhibition of STRA6/JAK2/STAT3 pathway either by specific siRNAs or inhibitors could decrease the expression of Atrogin‐1 and MuRF1 (>50%) and decrease the expression of MyoD and MyoG (>3‐fold) in holo‐RBP4‐treated C2C12 myotube. RBP4 specific pharmacological antagonist A1120 significantly inhibited the activation of STRA6/JAK2/STAT3 pathway, ameliorated ectopic fat infiltration and protected against denervation‐induced muscle atrophy (30% increased myofibre cross‐sectional area) in mice.

**Conclusions:**

In conclusion, our data reveal that RBP4 promotes fat infiltration and muscle atrophy through a STRA6‐dependent and JAK2/STAT3 pathway‐mediated mechanism in denervated skeletal muscle. Our results suggest that lowering RBP4 levels might serve as a promising therapeutic approach for prevention and treatment of muscle atrophy.

## Introduction

Muscle fat infiltration, characterized by increased deposit of lipid in skeletal muscle, has been considered as an irreversible pathological condition that is closely correlated with various skeletal muscle disorders.^1^ Although muscles are highly regenerative tissues following acute injury; however, continued fat infiltration under chronic pathological stimulus often results in accumulation of connective tissue and detrimental fibrosis in skeletal muscle system.^2^ In the old adults, increased fat infiltration promotes the reduction of muscle mass and impairs muscle function, leading to a higher susceptibility for sarcopenia.^3^ In the skeletal muscle, deposition of lipids and their derivatives not only induces lipotoxicity, which causes mitochondrial dysfunction, oxidative stress, insulin resistance and inflammation but also enhances the secretion of a variety of adipokines, which may aggravate muscle mass loss and disability.^4^

Retinol‐binding protein 4 (RBP4) is an approximately 21‐kDa protein that transports retinol (vitamin A) in circulation.^5^ RBP4 has also been well known as an important adipokine that contributes to diabetes and obesity through its pro‐inflammatory effect.^6^, ^7^, ^8^ Increased secretion of RBP4 impairs the glucose uptake of skeletal muscle which in turn causes insulin resistance.^9^ We previous showed that serum RBP4 was elevated in patients with sarcopenia and correlated with the decline of muscle mass and physical dysfunction.^10^ Moreover, the expression of RBP4 was also detected in skeletal muscle,^11^, ^12^ suggesting a potential RBP4‐mediated cross‐talk between adipose tissue and skeletal muscle in the pathogenesis of sarcopenia. Therefore, the aim of the present study was to investigate the effects of RBP4 on muscle fat infiltration and muscle atrophy as well as the underlying mechanisms in a mouse model of denervation‐induced muscle atrophy.

## Materials and methods

### Animals, denervation protocol and tissue collection

RBP4 knockout mice (Strain No.T012705) with the background of C57/BL6J were generated by GemPharmatech Co. Ltd. (Nanjing, China) by using CRISPR/Cas9 technology. The exon1‐exon5 of Rbp4‐201 (ENSMUST00000025951.13) transcript which contains start codon ATG was selected as the knockout region. The deletion was confirmed by genotyping (*Figure* *S1*). C57/BL6J wild‐type and RBP4 knockout male mice of 8 weeks old were housed at the temperature of 24 ± 2°C and the humidity of 45 ± 15% with a 12 h light/dark cycle. For denervation procedure, mice were anaesthetised with 3.0% isoflurane gas in pure oxygen and a small incision (≤0.5 mm) was made in the right tight skin. Muscle packages from gluteal and biceps femoris muscles were carefully separated by cutting the facia. The sciatic nerve was cut and a small (2–5 mm) section was removed to prevent reinnervation. Denervation was performed unilaterally, using the contralateral hindlimb as a control. Gastrocnemius, tibialis anterior muscle, soleus muscle and extensor digitorum longus were dissected from denervated and contralateral hindlimbs at 3, 7, 14 and 28 days after denervation surgery. Muscle samples were frozen and stored at −80°C until processing. All animal protocols were in accordance with the ethical standards laid down in the 1964 Declaration of Helsinki and its later amendments and approved by the Institutional Animal Care and Use Committee of Nanjing Medical University (permit number: IACUC‐1905027).

### Intramuscular injection of recombinant RBP4

Mouse His‐RBP4 (Sino Biological, China) was produced in HEK293 Cells and purified by Ni‐nitrilotriacetic acid‐agarose beads and high‐performance liquid chromatography with the endotoxin level <1.0 EU per microgram of the protein. For intramuscular injection, the gastrocnemius or tibialis anterior muscles was intramuscular injected with 50 μL of Retinol‐free RBP4 (apo‐RBP4, 100 μg/mL) or Retinol‐bound RBP4 (holo‐RBP4, 100 μg/mL) per day for 14 days. The control group was injected with the same amount of normal saline.

### Treatment with RBP4 inhibitors

A1120 (MCE, USA), a non‐retinoid RBP4 antagonist,^13^ was oral gavage administered with different concentrations (10, 30, 50 mg/kg) per day for 14 days to 8‐week‐old wild‐type mice. Denervation was performed after the first treatment, and muscles were collected 14 days after denervation.

### Immunofluorescence and myotube diameter measurement

Cryosections (6 μm) of skeletal muscles were fixed in 4% paraformaldehyde for 20 min, permeabilized with 0.5% Triton X‐100 for 15 min, and then blocked with 5% bovine serum albumin (BSA) for 1 h and incubated overnight at 4°C with anti‐RBP4 (1:200, ab109193, Abcam, UK) and anti‐laminin (1:300, L9393, Sigma‐Aldrich, USA). Cy3‐AffiniPure Rabbit Anti‐Mouse IgG (H + L) (1:500, 711‐165‐152, Jackson ImmunoResearch, USA) and Cy™3 AffiniPure Donkey Anti‐Mouse IgG (H + L) (1:500, 715‐165‐150, Jackson ImmunoResearch, USA) secondary antibody were added to visualize the staining. 4′,6‐diamidino‐2‐phenylindole (DAPI, 1 μg/mL, Sigma‐Aldrich, USA) was used to counterstain the nuclei. Images were captured using fluorescence microscope (Zeiss Axio Scope, Germany). Fibre diameter and cross‐sectional area measurements were carried out by exploiting the ROI manager plugin from the ImageJ software (National Institutes of Health, USA). Quantifications were performed using 3–7 images per muscle and 2–3 muscles per condition.

### Haematoxylin–eosin staining

Fresh skeletal muscle samples were fixed with 4% paraformaldehyde overnight, and embedded with paraffin, serially sliced to 4 μm for haematoxylin–eosin (HE) staining. For muscle fibre cross‐sectional area analysis, images were captured using microscope (Zeiss Axio Scope.A1, Germany) and calculated by using the ImageJ software (National Institutes of Health, USA) in five random fields of each section.

### Nile red staining

To visualize the lipid accumulation, skeletal muscle samples were fixed in 4% paraformaldehyde for 15 min and then stained with Nile Red (Fluka) at a concentration of 10 μg/mL and Alexa 633 Phalloidin (Life Technology) overnight, washed four times with phosphate‐buffered saline (PBS) for 15 min each, and mounted in VECTASHIELD Mounting Medium (Vector Laboratories).

### Immunofluorescence staining

Immunofluorescence staining on frozen skeletal muscle sections was performed as described previously.^14^ Briefly, after incubation with anti‐RBP4 (1:200, Abcam, UK) overnight at 4°C, Alexa Fluor® 488 AffiniPure Donkey Anti‐Rabbit IgG (H + L) (1:200, Jackson ImmunoResearch, USA) was added to visualize the staining. DAPI (1 μg/mL, Sigma, USA) was used to counterstain the nuclei. The staining was observed and quantified in 10 randomly selected 10 areas of each sample using a fluorescence microscope with Cellsens Dimention 1.15 software (Olympus, Japan).

### Retinol and retinol derivatives measurements

Gastrocnemius retinol, retinal, retinyl ester and retinoic acid levels were examined by liquid chromatography–mass spectrometry (LC–MS). Briefly, gastrocnemius was homogenized in ice‐cold PBS with a motorized homogenizer (Thermo Fisher Scientific, USA). An aliquot of 150 μL homogenates was treated with an equal volume of acetonitrile and vortexed vigorously. Then 900 μL of pre‐cooled *tert*‐butyl methyl ether was added and vortexed. After standing at −20°C for 1 h, the solution was centrifuged at 4°C, 13 500 *g* for 20 min. The supernatant was evaporated to dryness and re‐suspended in 1 mL of methanol prior to injection. An aliquot was injected into a 5500 QTRAP LC–MS/MS instrument with APCI ionization (Applied Biosystems, USA). The autosampler was cooled to 4°C, and samples were placed in the closed compartment only immediately prior to injection. Analytes were separated using the following gradient protocol (flow, 400 μL/min; gradient, 0–0.1 min, 10% A and 90% B; 1–5 min, 45% A and 55% B; 5–6 min, 60% A and 40% B; 6–7.7 min, 60% A and 40% B; 7.7–7.8 min, 10% A and 90% B; 7.8–10 min, 5% A and 95% B). Mobile phase A: 5% acetonitrile, 20 mmol/L ammonium acetate and mobile phase B: 100% acetonitrile. Quantification was performed by interpolation and linear least‐squares regression using Skyline software (China).

### C2C12 myoblasts culture and differentiation

Murine C2C12 myoblasts were obtained from ATCC and incubated at 37°C, 5% CO_2_ in DMEM with 80 U/mL penicillin and 0.08 mg/mL streptomycin and 10% fetal bovine serum (Gibco, USA). For the induction of differentiation into myotubes, sub‐confluent myoblasts were switched to DMEM containing 2% horse serum (Biological Industries, Israel) and then cultured for 4–6 days.

### Treatment of C2C12 myotubes

Mature myotubes were treated with different concentrations of apo‐RBP4 (50, 100 and 150 μg/mL) or holo‐RBP4(50, 100 and 150 μg/mL) for 24 h. For inhibiting the stimulated by retinoic acid 6 (STRA6)/Janus kinase 2 (JAK2)/Signal transducer and activator of transcription 3 (STAT3) pathway, C2C12 myotubes were transfected with 50 nM siRNAs targeting STRA6 or STAT3 (RiboBio Co., Ltd., China) or treated with 10 μM JAK2 inhibitor AG490 (MCE, USA) for 24 h following treatment with holo‐RBP4. The siRNA sequences were listed in *Table* *S1*.

### Myotube diameter measurement

C2C12 myotubes were fixed with 4% paraformaldehyde for 20 min, permeabilized with 0.5% Triton X‐100 for 15 min, and then blocked with 5% BSA for 1 h. Cells were incubated with anti‐MHC (1:200, MF20, DSHB, USA) overnight at 4°C, then incubated with secondary antibody Cy3‐AffiniPure Rabbit Anti‐Mouse IgG (H + L) (1:500, Jackson ImmunoResearch, USA). Nuclear counterstaining was performed with DAPI. Images were captured using fluorescence microscope (Zeiss Axio Scope, Germany). Myotube diameter analysis was performed as previously published.^15^

### Isolation of total RNA and real‐time PCR analysis

Total RNA from skeletal muscles or C2C12 myotubes samples was isolated by Total RNA Extraction Kit (TIANGEN, China). cDNA was synthesized using PrimeScript™ RT reagent Kit (Perfect Real Time) (Takara, Japan). Quantitative real‐time PCR was performed using Maxima SYBR Green/ROX qPCR Master Mix (2X) (Thermo Scientific, USA) on LightCycler480 system (LightCycler, USA). The relative gene expression levels were calculated by the 2^−ΔΔCt^ method using glyceraldehyde 3‐phosphate dehydrogenase (GAPDH) as an internal control. All the primer sequences were listed in *Table* *S2*.

### Protein extraction and Western blot analysis

Skeletal muscles or myotubes samples were lysed with cold RIPA buffer (Beyotime Biotechnology, China) containing 1 mM NaF, 1 mM sodium orthovanadate and 1 mM phenylmethylsulfonyl fluoride. Equal amount of protein was separated by 10% SDS‐PAGE (Yeasen, China), transferred to PVDF membranes (Millipore, USA), and then blocked with 5% nonfat milk. Membranes were incubated with specific primary antibody overnight at 4°C. The following primary antibodies were used: anti‐RBP4 (Abcam, ab109193, UK), anti‐GAPDH (Proteintech, 60004‐1‐Ig, China), anti‐muscle atrophy F‐box (Atrogin‐1) (Abcam, ab168372, UK), anti‐muscle ring finger 1 (MuRF1) (R&D, AF5366, USA), anti‐myogenic differentiation 1 (MyoD) (Santa Cruz Biotechnology, sc‐32758, USA), anti‐myogenin (MyoG) (Developmental Studies Hybridoma Bank, F5D, USA), anti‐Proliferating cell nuclear antigen (PCNA) (Cell Signaling Technology, 13110, USA), anti‐Cyclin D1 (CCND1) (Cell Signaling Technology, 55506, USA), anti‐Bax (Abcam, ab32503, UK), anti‐Bcl‐2 (Abcam, ab182858, UK), anti‐stimulated by retinoic acid 6 (STRA6) (Proteintech, 22001‐1‐AP, China), anti‐p‐JAK2 (Cell Signaling Technology, 3771, USA), anti‐JAK2 (Cell Signaling Technology, 3230, USA), anti‐p‐STAT3 (Santa Cruz Biotechnology, sc‐8059, USA), anti‐STAT3 (Santa Cruz Biotechnology, sc‐8019, USA), anti‐p‐STAT5 (Cell Signaling Technology, 9359, USA) and anti‐STAT5 (Cell Signaling Technology, 25656, USA). The horseradish peroxidase conjugated secondary antibody was incubated for 1.5 h, then the immune complexes were detected by Immobilon Western HRP Substrate Peroxide Solution (Millipore, USA). Images were acquired using ChemiDocTM XRS + Imaging System (Bio‐Rad, USA). Band densitometry measurements were assessed using Image Lab 6.0 software.

### Statistical analysis

Data were presented as mean ± *SEM* and analysed by SPSS 21.0. Normality of distribution was assessed using the Kolmogorov–Smirnov test. Comparison between two groups was performed with Student's *t* tests or Mann–Whitney *U* tests. For comparison between more than two groups, one‐way ANOVA or two‐way ANOVA test with post‐hoc correlation was used as appropriate. Significance was accepted as *P* < 0.05.

## Results

### RBP4 expression is induced by denervation in mice

**Figure 1:**
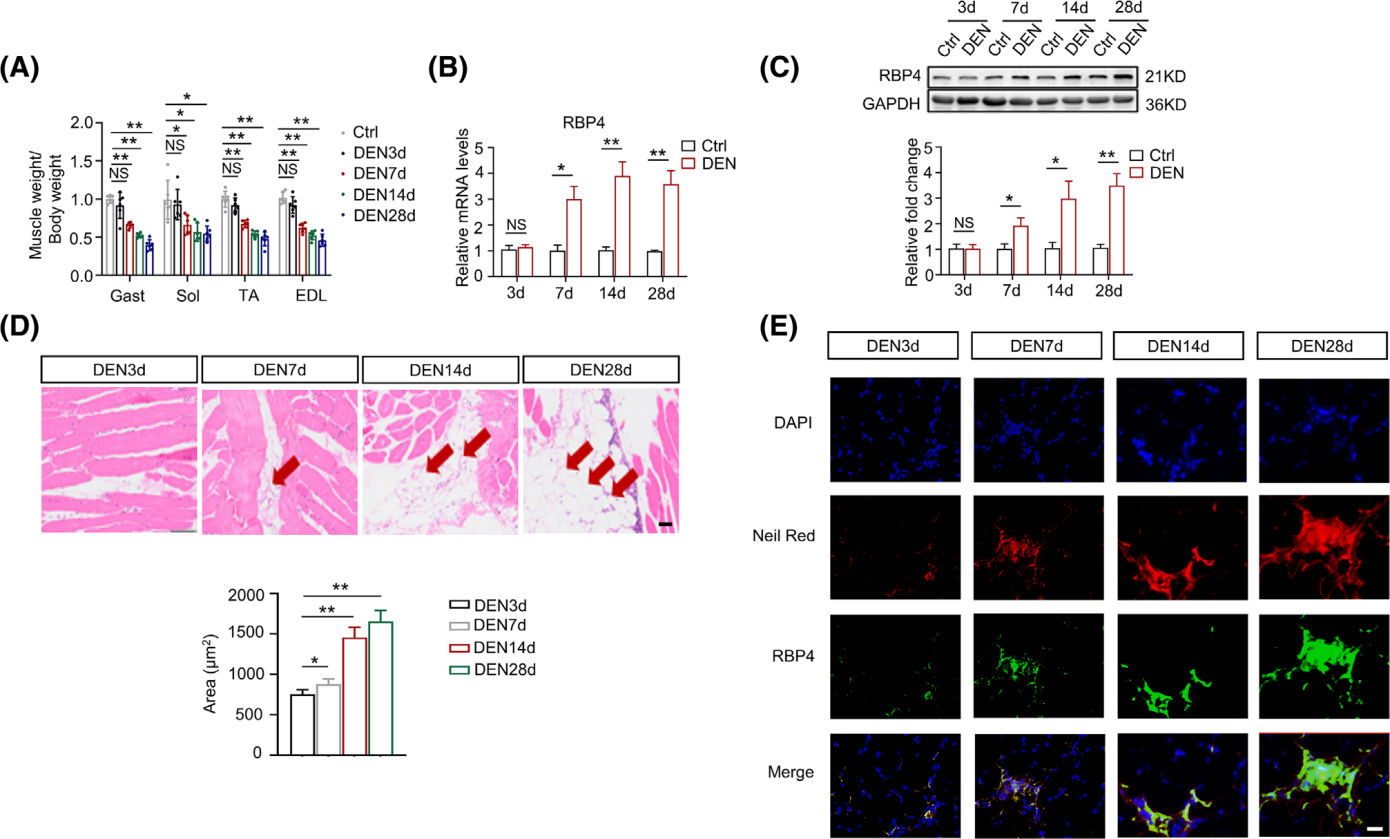
RBP4 expression is induced in denervated skeletal muscle in mice. Denervation‐induced muscle atrophy model was constructed in wild‐type mice. (*A*) Ratio of skeletal muscle to body weight. (*B–C*) The mRNA (*B*) and protein (*C*) levels of RBP4 in gastrocnemius. (*D*) Representative haematoxylin–eosin staining of myofibre cross‐section of gastrocnemius (upper) and area of infiltrated fat in gastrocnemius (lower). Arrow: Infiltrated fat. Scale bar = 50 μm. (*E*) Immunofluorescence staining for RBP4 (green) in infiltrated fatty region (red) in gastrocnemius. Scale bar = 100 μm. *n* = 8 per group, Ctrl, control; DEN, denervation; EDL, extensor digitorum longus; Gast, gastrocnemius; Sol, soleus muscle; TA, tibialis anterior muscle. For *Figure* *1* *A,D*, one‐way ANOVA analyses with post‐hoc correlation were used. For *Figure* *1* *B,C*, Student's *t* tests were used. *P < 0.05; **P < 0.01; NS, no significance.

To evaluate the possible involvement of RBP4 in the pathogenesis of muscle atrophy, we first examined RBP4 expression in the skeletal muscles of mice suffered from denervation. Skeletal muscle atrophy was confirmed by the decreased muscle weight (*Figure* *1* *A*), myofibre cross‐sectional area (*Figure* *S2A,B*), as well as the increased expression of Atrogin‐1 and MuRF1 (*Figure* *S1* *C*). In addition, the expression of myogenic regulators myogenic MyoD and MyoG was also significantly increased after denervation as compared with the control hindlimbs (Figure S2C). The mRNA and protein levels of RBP4 were significantly up‐regulated from 7 days and sustained for 28 days after denervation both in gastrocnemius (*Figure* *1* *B,C*) and tibialis anterior muscles (*Figure* *S3A,B*). As shown in *Figures* *1D* and S3C, denervation caused obvious fat accumulation in skeletal muscles. Interestingly, immunofluorescence analysis indicated that increased RBP4 expression was localized in the infiltrated fatty region (*Figures* *1E* and *S3D*). Taken together, these results suggest that the expression of RBP4 is induced by denervation procedure and might be associated with fatty infiltration.

### Knockout of RBP4 attenuates denervation‐induced skeletal muscle atrophy

**Figure 2:**
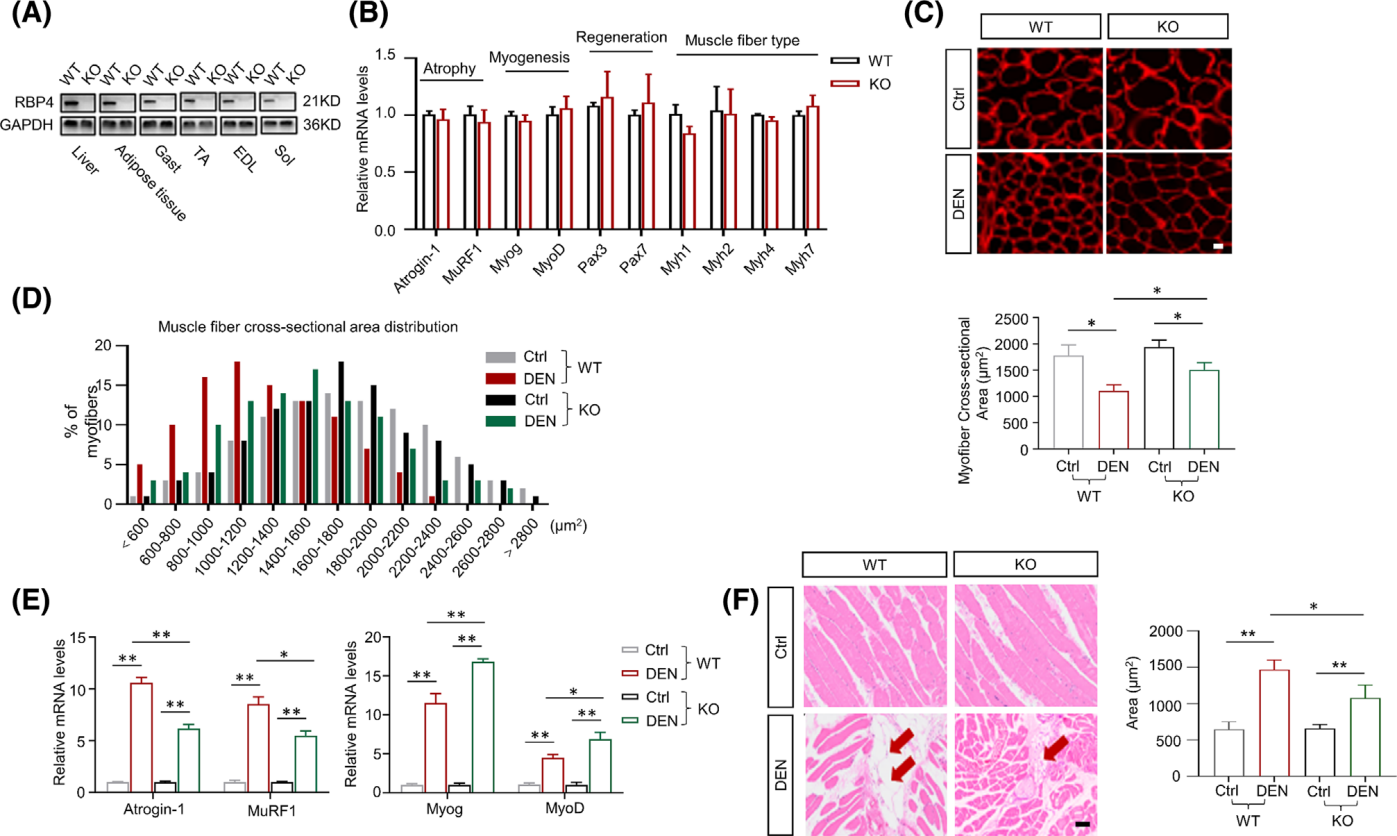
Knockout of RBP4 attenuates denervation‐induced skeletal muscle atrophy. RBP4 knockout mice were applied and subjected to denervation procedure. (*A*) RBP4 expression in different tissues of wild‐type and knockout mice. (*B*) Expression of genes related to muscle atrophy, myogenesis, muscle regeneration, and muscle fibre‐type transformation in gastrocnemius. (*C*) Representative immunofluorescence staining of myofibre with laminin (red) in gastrocnemius (upper) and myofibre cross‐sectional area of gastrocnemius (lower). Scale bar = 50 μm. (*D*) Distribution of myofibre with different cross‐sectional area in gastrocnemius. (*E*) The mRNA levels of muscle atrophy marker Atrogin‐1 and MuRF1, and myogenic regulator MyoD and MyoG in gastrocnemius. (*F*) Representative haematoxylin–eosin staining of myofibre cross‐section of gastrocnemius (left) and area of infiltrated fat in gastrocnemius (right). Arrow: Infiltrated fat. Scale bar = 50 μm. *n* = 8 per group, Ctrl, control; DEN, denervation; EDL, extensor digitorum longus; Gast, gastrocnemius; KO, knockout; Sol, soleus muscle; TA, tibialis anterior muscle; WT, wild type. For *Figure* *2* *B*, Mann–Whitney *U* tests were used. For *Figure* *2* *C,E,F*, two‐way ANOVA analyses with post‐hoc correlation were used. *P < 0.05; **P < 0.01.

To further investigate the effects of RBP4 in skeletal muscle atrophy, RBP4 knockout mice were applied and subjected to denervation procedure. In gastrocnemius muscle, knockout of RBP4 *per se* (*Figure* *2A*) did not affect the expression levels of genes related to muscle atrophy, myogenesis, muscle regeneration and muscle fibre type transformation (*Figure* *2B*). However, denervation‐induced muscle atrophy was attenuated in RBP4 knockout mice (*Figure* *2C,D*), together with decreased expression levels of atrophy marker Atrogin‐1 and MuRF1, as well as increased expression levels of myogenesis regulators MyoD and MyoG (*Figure* *2* *E*). Moreover, denervation‐induced fatty infiltration was also ameliorated in RBP4 knockout mice (*Figure* *2* *F*). Similar results were observed in tibialis anterior muscle of RBP4 knockout mice (*Figure* *S4*). These results indicate that knockout of RBP4 can attenuate denervation‐induced skeletal muscle atrophy and fat infiltration.

### RBP4 aggregates denervation‐induced skeletal muscle atrophy

**Figure 3:**
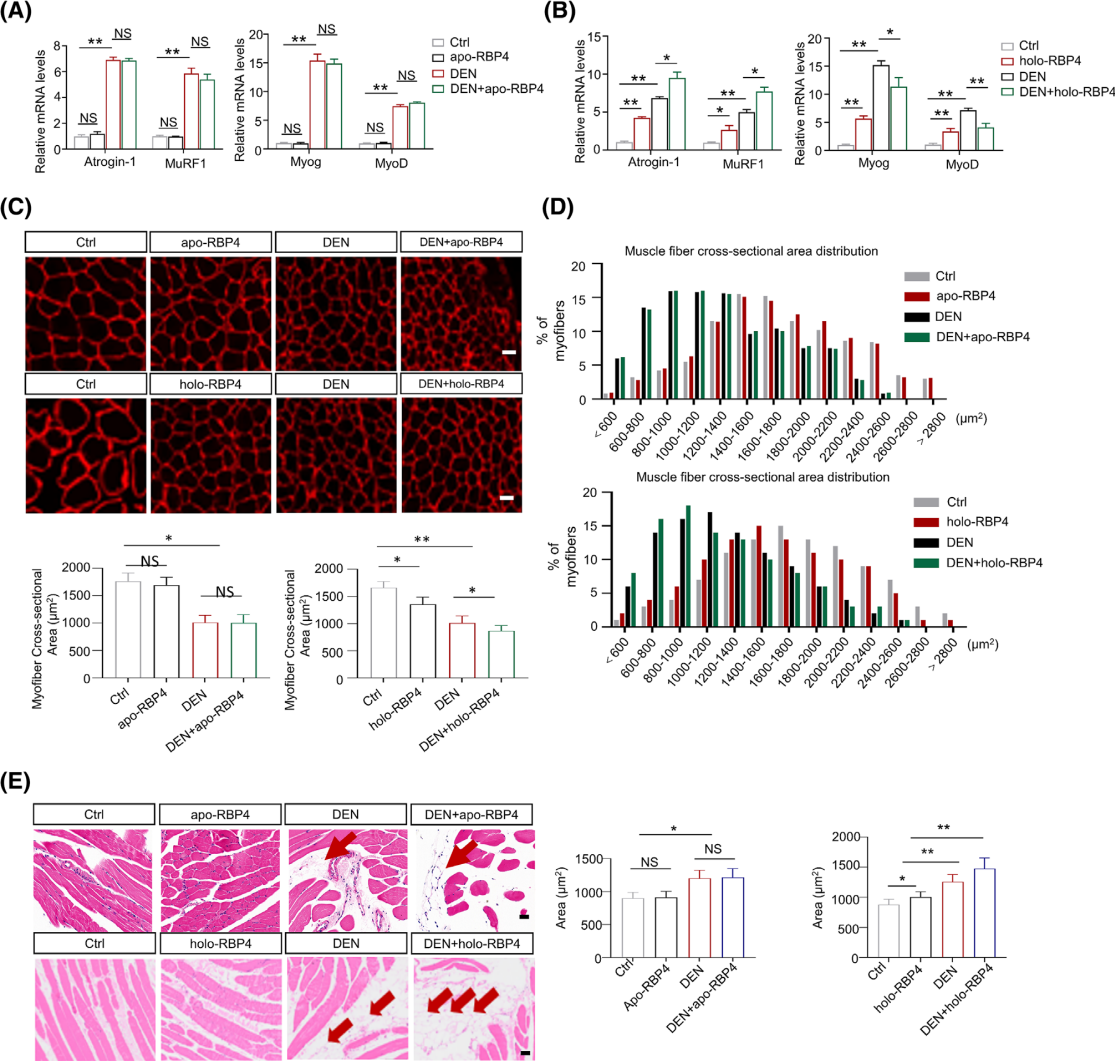
RBP4 aggregates denervation‐induced skeletal muscle atrophy in mice. RBP4 knockout mice were subjected to denervation and then received the injection of either retinol‐free RBP4 (apo‐RBP4) or retinol‐bound RBP4 (holo‐RBP4) before sacrifice. (*A*) The mRNA levels of muscle atrophy marker Atrogin‐1 and MuRF1, and myogenic regulator MyoD and MyoG in gastrocnemius from mice treated with apo‐RBP4. (*B*) The mRNA levels of muscle atrophy marker Atrogin‐1 and MuRF1, and myogenic regulator MyoD and MyoG in gastrocnemius from mice treated with holo‐RBP4. (*C*) Representative immunofluorescence staining of myofibre with laminin (upper) and myofibre cross‐sectional area (lower) of gastrocnemius from mice treated with apo‐RBP4 and holo‐RBP4. Scale bar = 50 μm. (*D*) Distribution of myofibre with different cross‐sectional area in gastrocnemius from mice treated with apo‐RBP4 (upper) and holo‐RBP4 (lower). (*E*) Representative haematoxylin–eosin staining of myofibre cross‐section of gastrocnemius from mice treated with apo‐RBP4 and holo‐RBP4. Arrow: Infiltrated fat. Scale bar = 50 μm. *n* = 8 per group, Ctrl, control; DEN, denervation. One‐way ANOVA analyses with post‐hoc correlation were used. *P < 0.05; **P < 0.01; NS, no significance.

We next examined the effects of RBP4 treatment on denervation‐induced skeletal muscle atrophy. RBP4 knockout mice were subjected to denervation and then received the injection of either retinol‐free RBP4 (apo‐RBP4) or retinol‐bound RBP4 (holo‐RBP4) before sacrifice. Denervation operation caused striking increase in the expression of Atrogin‐1, MuRF1, MyoD and MyoG; however, no significant changes were observed after treatment with apo‐RBP4 in the gastrocnemius of RBP4 knockout mice (*Figure* *3* *A*). By contrast, treatment with holo‐RBP4 induced a profound increase in the expression of Atrogin‐1, MuRF1, MyoD and MyoG both in the sham and injured hindlimbs (*Figure* *3* *B*). Moreover, muscle atrophy was also aggregated in holo‐RBP4 treated mice, as evidenced by decreased myofibre cross‐sectional area (*Figure* *3* *C,D*), along with more dramatic ectopic fat accumulation (*Figure* *3* *E*). No significant change of muscle atrophy (*Figure* *3* *C,D*) and fat infiltration (*Figure* *3* *E*) was observed in apo‐RBP4 treated mice. The detrimental effects of holo‐RBP4 on denervation‐induced muscle atrophy were also displayed in the tibialis anterior muscle of RBP4 knockout mice (*Figure* *S5*).

### RBP4 induces muscle atrophy in C2C12 myotubes

**Figure 4:**
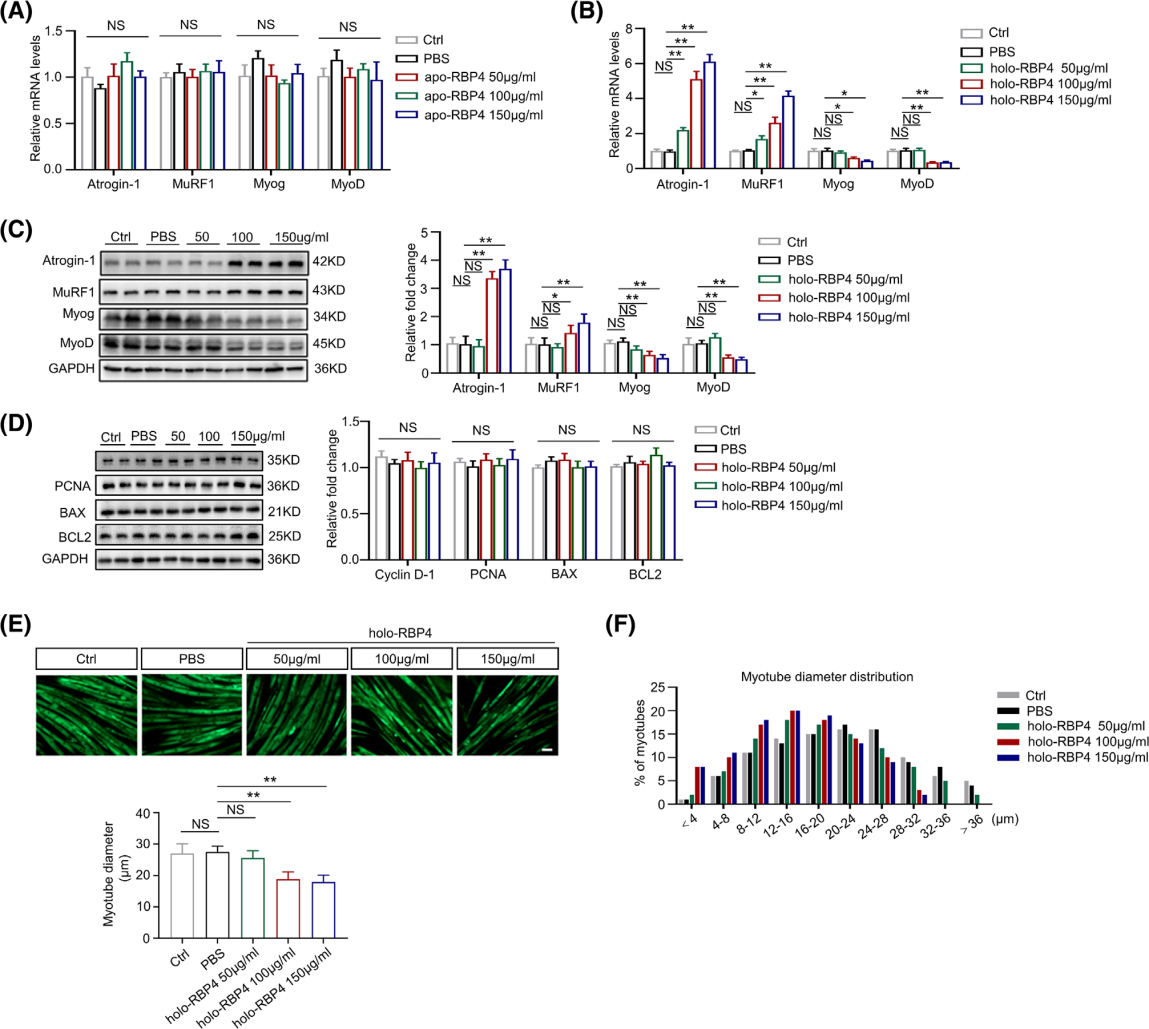
RBP4 induces muscle atrophy in C2C12 myotubes. C2C12 myoblasts were incubated with differentiation medium until cell fusion and then treated with retinol‐free RBP4 (apo‐RBP4) or retinol‐bound RBP4 (holo‐RBP4). (*A*) The mRNA levels of muscle atrophy marker Atrogin‐1 and MuRF1 as well as myogenic regulator MyoD and MyoG in C2C12 myotubes treated with apo‐RBP4. (*B*) The mRNA levels of muscle atrophy marker Atrogin‐1 and MuRF1 as well as myogenic regulator MyoD and MyoG in C2C12 myotubes treated with holo‐RBP4. (*C*) The protein levels of muscle atrophy marker Atrogin‐1 and MuRF1 as well as myogenic regulator MyoD and MyoG in C2C12 myotubes treated with holo‐RBP4. (*D*) The protein levels of proliferation marker PCNA and CCND1 as well as apoptosis marker Bax and Bcl‐2 in C2C12 myotubes treated with holo‐RBP4. (*E*) Representative immunofluorescence staining of myotubes with MHC (green, upper) and myotube diameters of C2C12 myotubes treated with holo‐RBP4. Scale bar = 50 μm. (*F*) Distribution of myotube with different diameter in C2C12 myotubes treated with holo‐RBP4. *n* = 6 per group, Ctrl, control. One‐way ANOVA analyses with post‐hoc correlation were used. *P < 0.05; **P < 0.01; NS, no significance.

To further confirm the effect of RBP4 on muscle atrophy *in vitro*, C2C12 myoblasts were incubated with differentiation medium until cell fusion (*Figure* *S6A*) and then treated with apo‐RBP4 or holo‐RBP4. Consistently, apo‐RBP4 treatment had no effect on the expression of Atrogin‐1, MuRF1, MyoD and MyoG (*Figure* *4* *A*). By contrast, the expression levels of Atrogin‐1and MuRF1 were increased whereas the expression levels of MyoD and MyoG were decreased after holo‐RBP4 treatment (*Figure* *4* *B,C*). In addition, holo‐RBP4 treatment also promoted the apoptosis of C2C12 myotubes, as evidenced by increased expression of Bax and decreased expression of Bcl‐2, without any effect on the proliferation markers PCNA and CCND1 (*Figure* *4* *D*). Moreover, holo‐RBP4 stimulation had a dose‐dependent effect on the reduction of C2C12 myotube diameter (*Figure* *4* *E,F*). These results indicate that holo‐RBP4 can also induce muscle atrophy in C2C12 myotubes.

### RBP4 induces muscle atrophy through activating STRA6

**Figure 5:**
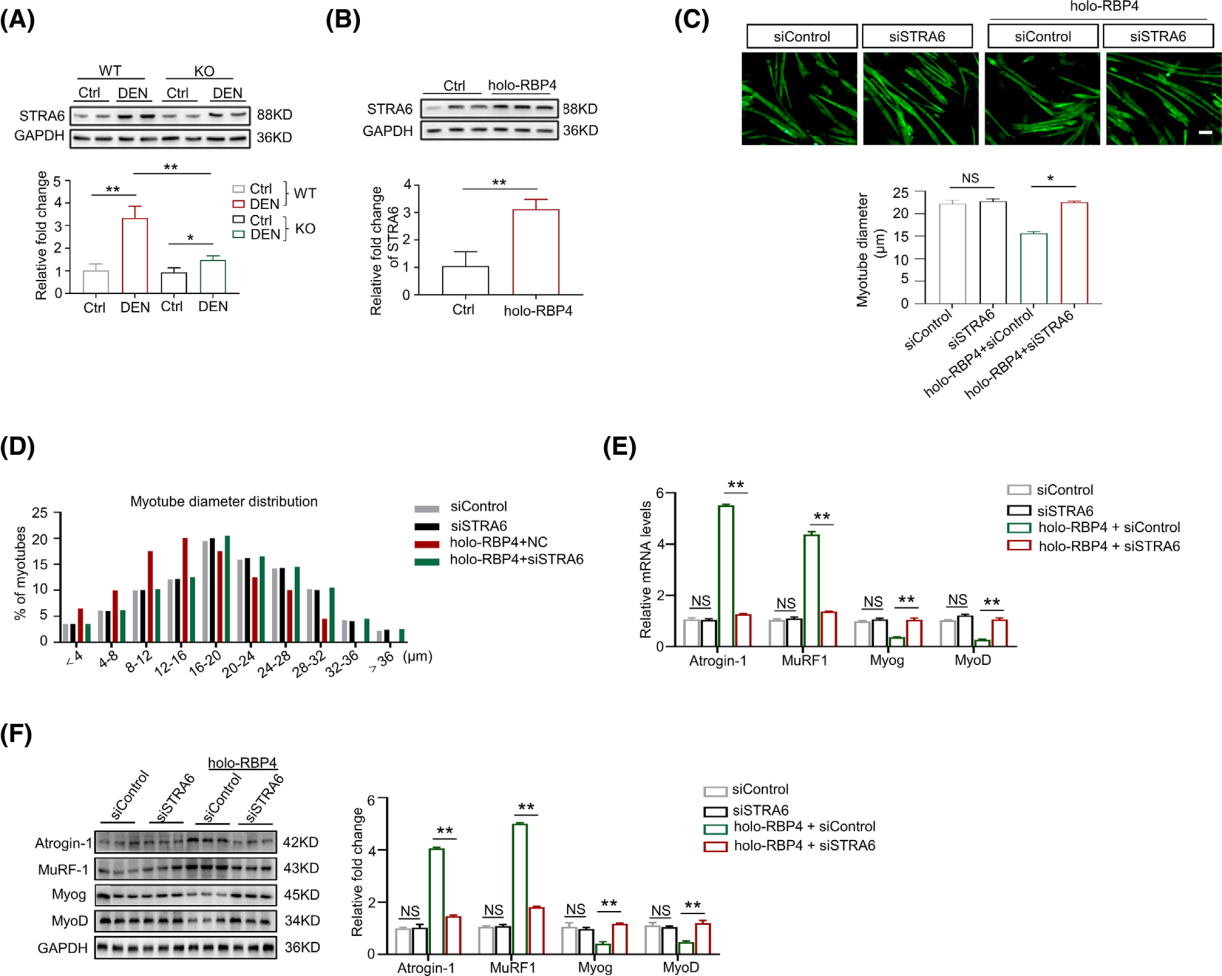
RBP4 induces muscle atrophy through activating STRA6. (*A*) The protein level of STRA6 in gastrocnemius from wild‐type and RBP4 knockout mice. (*B*) The protein level of STRA6 in C2C12 myotubes treated with holo‐RBP4. (*C*) Representative immunofluorescence staining of myotubes with MHC (green, upper) and myotube diameters (lower) of C2C12 myotubes treated with holo‐RBP4 combined with siSTRA6. Scale bar = 50 μm. (*D*) Distribution of myotube with different diameter in C2C12 myotubes treated with holo‐RBP4 combined with siSTRA6. (*E*) The mRNA levels of muscle atrophy marker Atrogin‐1 and MuRF1 as well as myogenic regulator MyoD and MyoG in C2C12 myotubes treated with holo‐RBP4 combined with siSTRA6. (*F*) The protein levels of muscle atrophy marker Atrogin‐1 and MuRF1 as well as myogenic regulator MyoD and MyoG in C2C12 myotubes treated with holo‐RBP4 combined with siSTRA6. *n* = 8 per group for mice and *n* = 8 per group for C2C12 myotubes. Ctrl, control; DEN, denervation; KO, knockout; WT, wild type. For *Figure* *5* *A,C,E,F*, one‐way ANOVA analyses with post‐hoc correlation were used. For *Figure* *5* *B*, Mann–Whitney *U* tests were used. *P < 0.05; **P < 0.01; NS, no significance.

Previous studies have identified that STRA6 is the high‐affinity cell‐surface receptor of RBP4 which functions in the uptake of retinol from holo‐RBP4,^16^ while toll‐like receptors 2 (TLR2) and 4 (TLR4) contribute to its pro‐inflammatory effect.^8^ We therefore examined which receptor mediates RBP4‐induced muscle atrophy. There was no significant difference of the expression of TLR2 and TLR4 between RBP4 knockout and wild‐type mice after denervation (*Figure* *S6B*). In addition, although denervation increased the expression of proinflammatory factors including interleukin‐1β (IL‐1β), IL‐6, tumour necrosis factor‐α, and monocyte chemoattractant protein‐1; however, no significant difference was observed between RBP4 knockout and wild‐type mice after denervation (*Figure* *S6C*). By contrast, denervation induced a significant increase of STRA6 expression, which was alleviated in RBP4 knockout mice (*Figures* *5* *A* and *S6B*). In C2C12 myotube, holo‐RBP4 treatment also increased the expression level of STRA6 (*Figures* *5* *B* and *S6D*). We next determined whether STRA6 is required for the induction of muscle atrophy by holo‐RBP4. Silencing of STRA6 by siRNA (*Figure* *S6E*) could ameliorate holo‐RBP4‐induced C2C12 myotube atrophy (*Figure* *5* *C,D*), down‐regulate the expression of Atrogen‐1 and MuRF1, but up‐regulate the myogenic markers MyoD and MyoG (*Figure* *5* *E,F*). These results indicate that STRA6‐dependent mechanism is involved in the promotion role of RBP4 in muscle atrophy.

### JAK2/STAT3 pathway mediates the STRA6‐dependent effect of RBP4 on muscle atrophy

**Figure 6:**
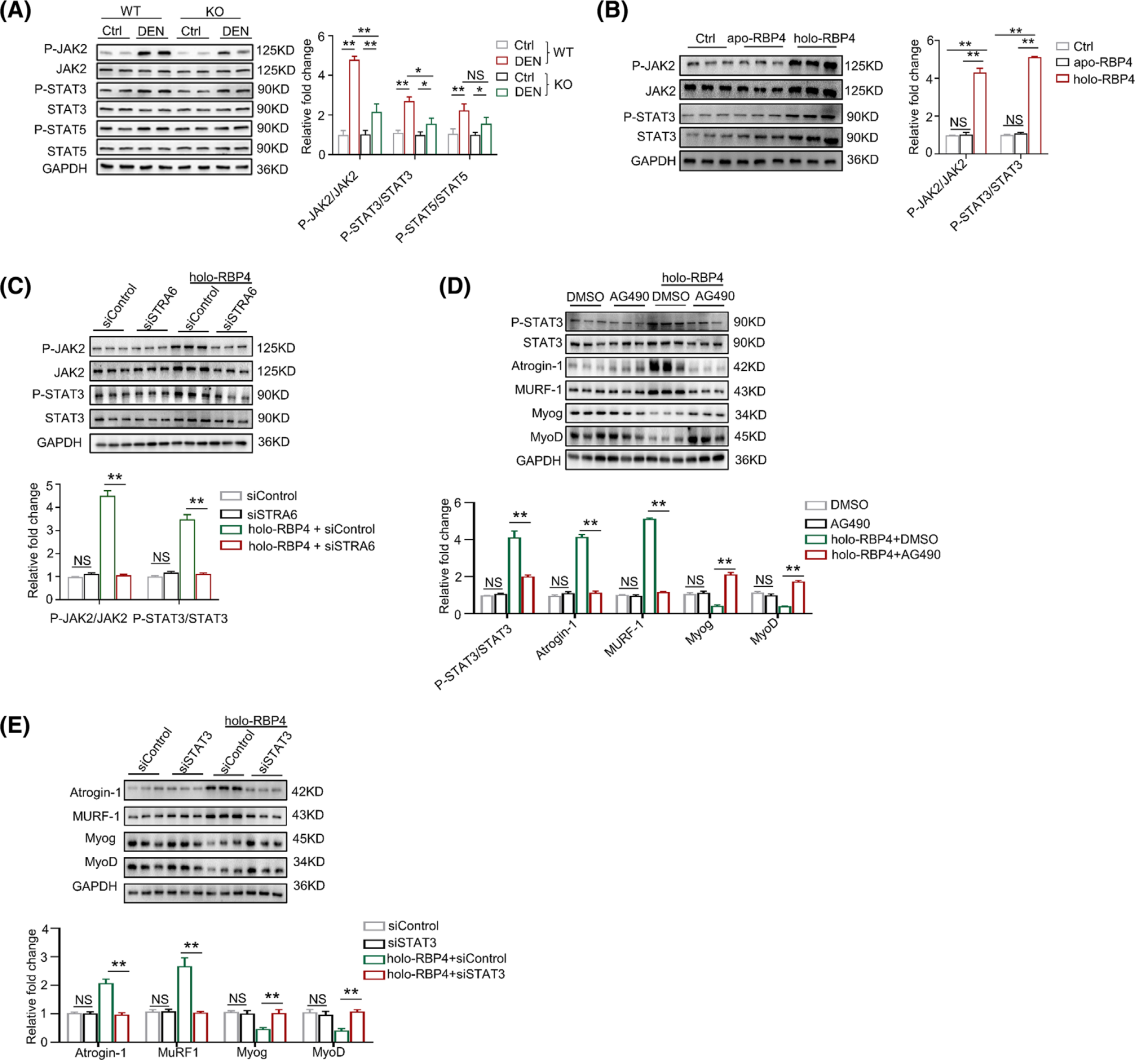
JAK2/STAT3 pathway mediates the STRA6‐dependent effect of RBP4 on muscle atrophy. (*A*) The protein levels of JAK2, STAT3 and STAT5 in gastrocnemius from wild‐type and RBP4 knockout mice. (*B*) The protein level of JAK2 and STAT3 in C2C12 myotubes treated with apo‐RBP4 and holo‐RBP4. (*C*) The protein level of JAK2 and STAT3 in C2C12 myotubes treated with holo‐RBP4 combined with siSTRA6. (*D*) The protein levels of STAT3, muscle atrophy marker Atrogin‐1 and MuRF1, as well as myogenic regulator MyoD and MyoG in C2C12 myotubes treated with holo‐RBP4 combined with AG490. (*E*) The protein levels of muscle atrophy marker Atrogin‐1 and MuRF1 as well as myogenic regulator MyoD and MyoG in C2C12 myotubes treated with holo‐RBP4 combined with siSTAT3. *n* = 8 per group for mice and *n* = 8 per group for C2C12 myotubes. Ctrl, control; DEN, denervation; KO, knockout; WT, wild type. For *Figure* *6* *A*, two‐way ANOVA analyses with post‐hoc correlation were used. For *Figure* *6* *B–E*, one‐way ANOVA analyses with post‐hoc correlation were used. *P < 0.05; **P < 0.01; NS, no significance.

Because retinol can enter the cells by membrane transport through STRA6,^16^ to investigate whether retinoids participate in the role of RBP4 in muscle atrophy, we first performed LC–MS to quantify the levels of retinoid isomers in the gastrocnemius from RBP4 knockout and wild‐type mice following denervation (*Figure* *S7*). Denervation did not cause any change of the levels of retinoids in skeletal muscles. After denervation, although the retinol level was lower in RBP4 knockout mice, the levels of retinal, retinyl ester, and retinoic acid remained unchanged (*Figure* *S7A–E*). Moreover, there was also no significant difference in the expression of key genes involved in cellular retinol metabolism in the skeletal muscle between RBP4 knockout and wild type mice (*Figure* *S7F*). A previous study has demonstrated that holo‐RBP4 can activate STRA6‐driven JAK signalling and downstream induction of STAT target genes.^17^ We then examined the expression levels of the members of JAK and STAT families in the skeletal muscle. Our qRT‐PCR found that only the mRNA levels of JAK2 (*Figure* *S8A,B*), STAT3 and STAT5 (*Figure* *S8C,D*) were increased after denervation and down‐regulated in RBP4 knockout mice. Further western blot showed that the active phosphorylation levels of JAK2, STAT3, and STAT5 were increased after denervation procedure; however, only phosphorylated JAK2 and STAT3 were lower in RBP4 knockout mice (*Figure* *6* *A*). We therefore focused on the JAK2/STAT3 pathway. Apo‐RBP4 treatment had no effect on the phosphorylation of JAK2 and STAT3 in C2C12 myotubes (*Figure* *6* *B*). By contrast, holo‐RBP4 treatment promoted the phosphorylation of JAK2 and STAT3 (*Figure* *6B*), which was inhibited by silencing of STRA6 (*Figure* *6C*), indicating that RBP4 activated JAK2/STAT3 pathway through STRA6. AG490, a specific JAK2 antagonist, could significantly inhibit the phosphorylation of JAK2 (*Figure* *S8E*). Moreover, the activation of STAT3‐increased expression of Atrogin‐1 and MuRF1, as well as decreased expression of MyoD and MyoG, were also ameliorated by AG940 treatment (*Figure* *6* *D*). Silencing of STAT3 by siRNA (*Figure* *S8F*) also had similar effects on RBP4‐induced changes of the expression of muscle atrophy markers and myogenic regulators (*Figure* *6* *E*) in C2C12 myotubes. Taken together, these results demonstrate that JAK2/STAT3 pathway is at least partially responsible for STRA6‐dependent effect of RBP4 on muscle atrophy.

### Pharmacological RBP4 inhibitor alleviates denervation‐induced muscle atrophy

**Figure 7:**
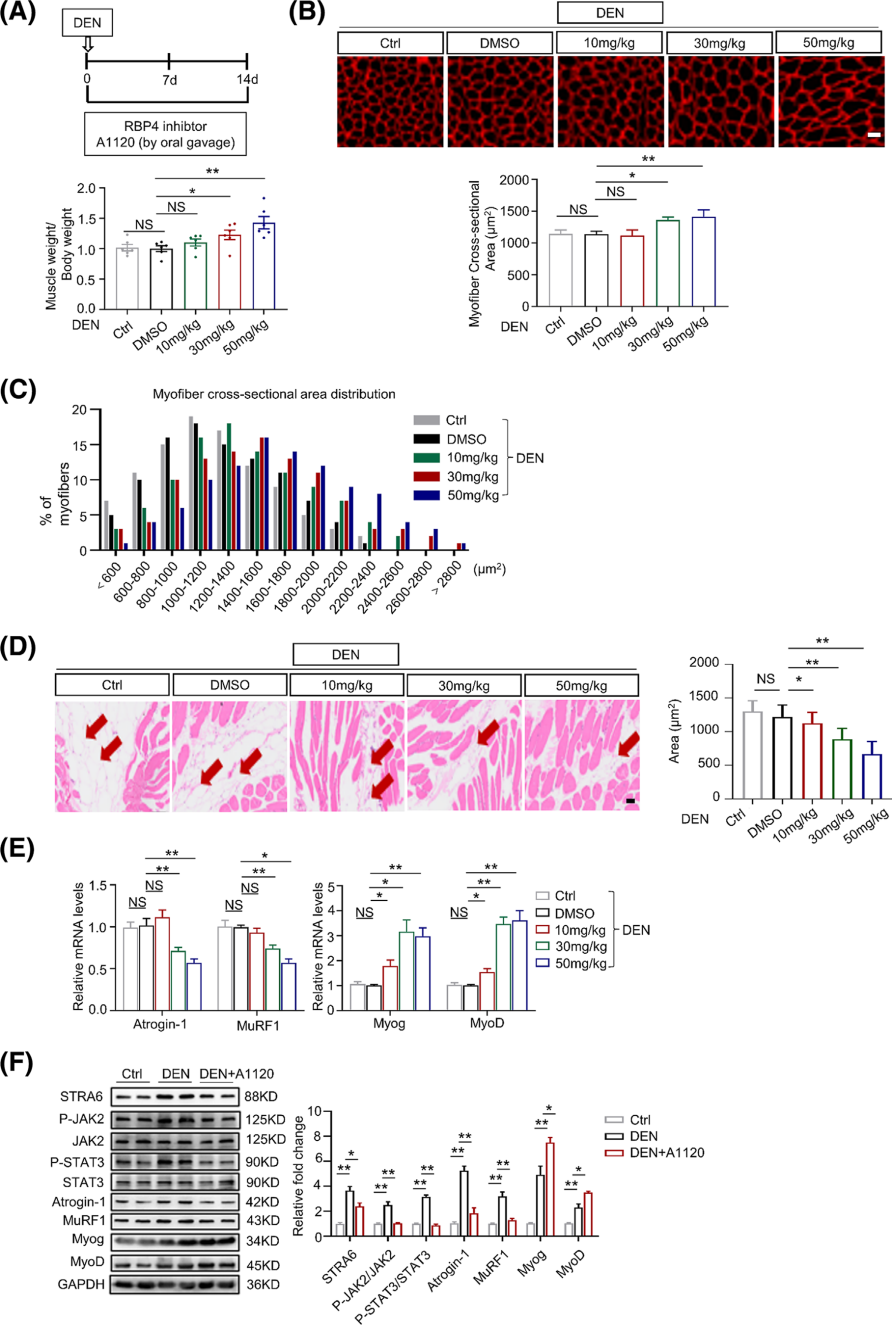
Pharmacological RBP4 inhibitor alleviates denervation‐induced muscle atrophy. (*A*) The ratio of muscle weight to body weight. (*B*) Representative immunofluorescence staining of myofibre with laminin (red, upper) and myofibre cross‐sectional area of gastrocnemius from mice treated with A1120. Scale bar = 50 μm. (*C*) Distribution of myofibre with different cross‐sectional area in gastrocnemius from mice treated with A1120. (*D*) Representative haematoxylin–eosin staining of myofibre cross‐section of gastrocnemius (left) and area of infiltrated fat in gastrocnemius (right) from mice treated with A1120. Arrow: Infiltrated fat. Scale bar = 50 μm. (*E*) The mRNA levels of muscle atrophy marker Atrogin‐1 and MuRF1 as well as myogenic regulator MyoD and MyoG in gastrocnemius from mice treated with A1120. (*F*) The protein level of STRA6, JAK2, STAT3, muscle atrophy marker Atrogin‐1 and MuRF1, as well as myogenic regulator MyoD and MyoG in gastrocnemius from mice treated with A1120. *n* = 8 per group, DEN, denervation. One‐way ANOVA analyses with post‐hoc correlation were used. *P < 0.05; **P < 0.01; NS, no significance.

To provide potential clinical proof‐of‐concept, we treated wild‐type mice subjected to denervation with the RBP4 specific inhibitor A1120^13^ for 14 days. Along with the increased muscle weight (*Figure* *7* *A* and *Table* *S3*), A1120 treatment with the dosage of 30 and 50 mg/kg alleviated denervation‐induced myofibre atrophy (*Figure* *7* *B,C*) and fat infiltration (*Figure* *7* *D*) in gastrocnemius muscles. Consistently, the expression levels of the muscle atrophy markers Atrogin‐1 and MuRF1 were decreased while the myogenic regulators MyoD and MyoG were increased after A1120 treatment (*Figure* *7* *E,F*). Moreover, A1120 injection also inhibited denervation‐induced activation of STRA6 and phosphorylation of JAK2/STAT3 pathway in denervated gastrointestinal muscle (*Figure* *7* *F*). Similar protective effects of A1120 on muscle atrophy were observed in the denervated tibialis anterior muscles (*Figure* *S10*). By contrast, no significant change of fat infiltration or muscle atrophy was found in the gastrocnemius (*Figure* *S9*) and tibialis anterior muscles (*Figure* *S11*) from sham hindlimbs.

## Discussion

**Figure 8:**
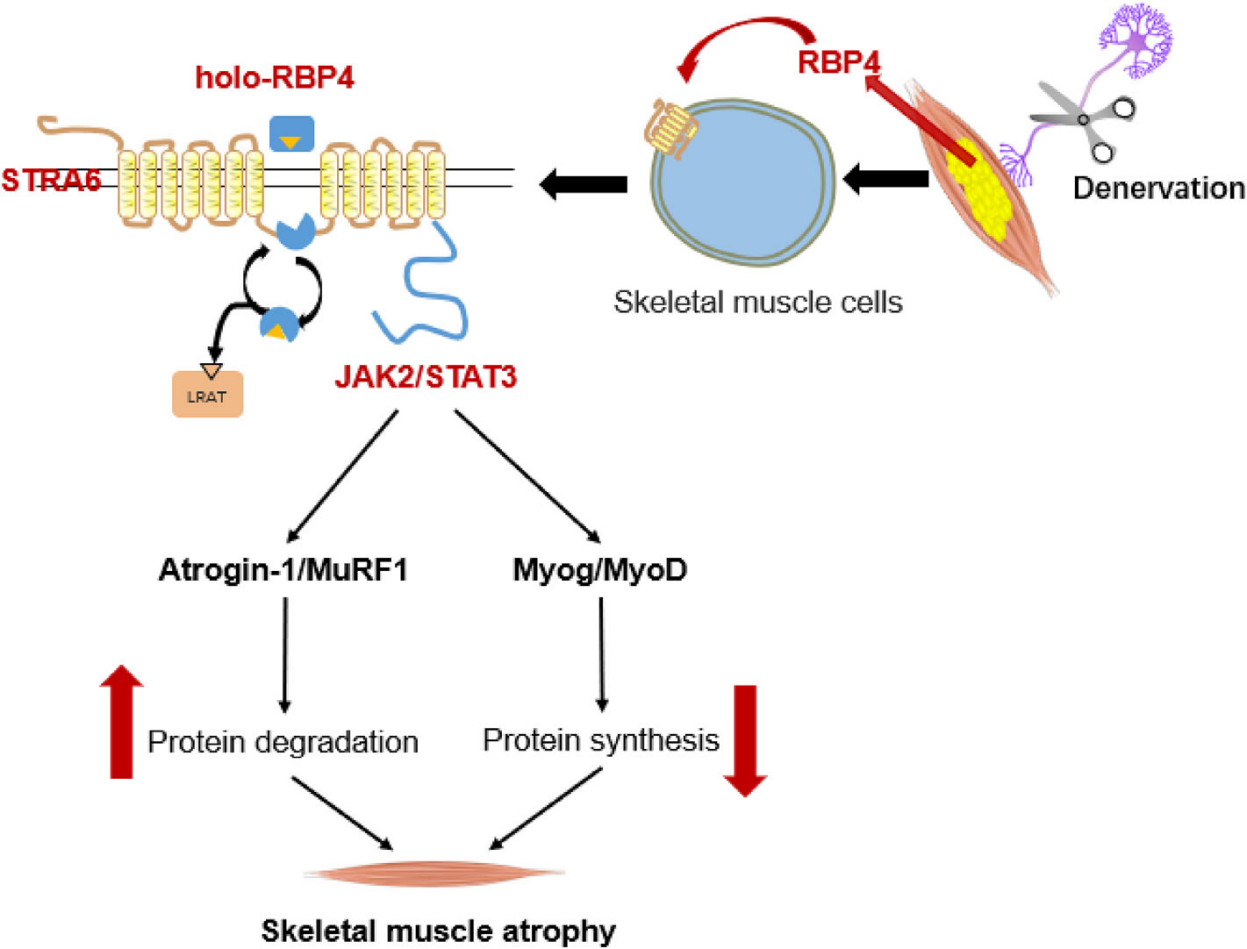
Schematic of the mechanism by which RBP4 promotes denervation‐induced skeletal muscle atrophy. During the development of denervation‐induced skeletal muscle atrophy, RBP4 expression is increased in the infiltrated adipose tissue, which in turn activates its membrane receptor STRA6 and promotes the phosphorylation of downstream JAK2 and STAT3, leading to increased protein degradation and decreased protein synthesis, which ultimately promotes muscle atrophy.

Our present study has identified a previously unrecognized pathological role of RBP4 as an adipokine that promotes denervation‐induced muscle atrophy (*Figure* *8*). We first showed that RBP4 expression was increased in the infiltrated fat in denervated skeletal muscle. Further *in vivo* experiments demonstrated that knockout of RBP4 alleviated while RBP4 injection exacerbated denervation‐induced fat infiltration and muscle atrophy. Mechanistically, RBP4 activated its membrane receptor STRA6 and promoted the phosphorylation of downstream JAK2 and STAT3, leading to increased protein degradation and decreased protein synthesis. Finally, pharmacological inhibition of RBP4 suppressed fat infiltration and protected against denervation‐induced muscle atrophy in mice. Therefore, our results suggest that lowering RBP4 might serve as a promising therapeutic approach for the prevention and treatment of muscle atrophy (sarcopenia).

Fat infiltration of skeletal muscle (myosteatosis) has been recognized as a common feature of many muscle disorders.^4^ As an endocrine organ, adipose tissue dynamically secrets a variety of bioactive hormones known as adipokines that may regulate the metabolism and function of the surrounding skeletal muscles through paracrine mechanism.^18^ Increasing numbers of human studies have indicated the association of circulating adipokines as a cross‐talk link between adipose tissue and skeletal muscle that contributes to the pathogenesis of sarcopenia.^19^ As an adipokine, RBP4 suppresses insulin‐stimulated phosphoinositide 3‐kinase activity and impairs insulin signalling in skeletal muscle.^9^ We previously showed that serum RBP4 was increased and correlated with the severity of sarcopenia in the old adults.^10^ We here demonstrated that RBP4 expression was increased along with the increased infiltrated fat in denervated skeletal muscle, which is supported by the colocalization of RBP4 and lipid droplets. The observed peak of RBP4 expression in Day 28 after denervation may therefore indicate increased synthesis and secretion of RBP4 by infiltrated adipose tissue. Deng et al.^20^ reported that adipose tissue can release RBP4‐rich exosome‐like vesicles which are taken up by monocytes, leading to further activation of macrophages. Our present study provides the first experimental evidence that RBP4 treatment exaggerated denervation‐induced muscle atrophy *in vivo* and promoted myotube atrophy *in vitro*. Therefore, RBP4 might also be released from ectopic adipose tissue via exosomes and plays a detrimental effect on denervated skeletal muscle. It is worth noting that previous studies have shown that overexpression of RBP4 suppresses the differentiation of preadipocytes and decelerates inguinal fat deposition via inhibiting insulin signalling^21^, ^22^ whereas knockdown of RBP4 promotes the adipogenesis in preadipocytes.^21^ Specifically, retinol‐bound holo‐RBP4 could block preadipocyte differentiation by activating retinoic acid receptor α (RARα) through retinol influx.^23^ By contrast, retinol‐free apo‐RBP4 triggers retinol efflux, leading to reduced RARα activity and enhanced adipogenesis in preadipocytes.^23^ These results might conflict with our present study because we found that RBP4 knockout ameliorated denervation‐induced fat infiltration in the skeletal muscle, which could be reversed by treatment of holo‐RBP4 but not apo‐RBP4. However, in human and mouse muscles, fibro‐adipogenic progenitors (FAPs) have been recognized as the prominent origin of intramuscular fat deposition that causes muscle loss.^3^ The significant differences in the biological characteristics between preadipocytes and FAPs might contribute to the heterogenous effects of holo‐RBP4 and apo‐RBP4 on adipogenesis in different regions. Nevertheless, whether the RBP4 synthesized in infiltrated adipose tissue influences adipogenesis in a paracrine or autocrine manner remains unclear. To better delineate its role in the intercellular cross‐talk between ectopic adipocytes and skeletal muscle cells, future studies in adipose‐specific RBP4 knockout mice are still needed.

The primary physiological function of RBP4 is to deliver retinol to target tissues via its specific membrane receptor STRA6.^24^ We here also showed that STRA6 was necessary for the detrimental effect of RBP4 on muscle atrophy. Considering previous studies have suggested that retinoic acid can stimulate myotube differentiation,^25^, ^26^ the role of RBP4 in muscle atrophy could be retinol dependent. However, although retinol level was lower in RBP4 knockout mice, no significant changes in the derived retinal, retinyl palmitate, and retinoic acid levels were observed in the skeletal muscles of RBP4 knockout mice. Despite functions as a vitamin A transporter, STRA6 can also serve as a cell surface cytokine receptor that activates JAK/STAT signalling cascade in response to holo‐RBP4.^27^ Previous studies have demonstrated that JAK2 can promote the phosphorylation of STAT3 which in turn causes dimerization and nuclear translocation of STAT3 where it functions as a transcription factor to increase the expression of skeletal muscle ubiquitin E3 ligases Atrogin‐1 and MuRF1.^28^ Detrimental activation of STAT3 has been directly linked to the loss of muscle mass in several models of muscle wasting, such as Duchenne muscular dystrophy, diabetes, chronic kidney disease and cancer cachexia^29^, ^30^ whereas genetic or pharmacological inhibition of STAT3 ameliorates muscle wasting in mice.^31^, ^32^, ^33^ Consistently, we found that holo‐RBP4 treatment promoted the phosphorylation of JAK2 and STAT3 while silencing of STRA6 suppressed holo‐RBP4‐induced activation of JAK2/STAT3 pathway in C2C12 myotubes. Importantly, inhibition of JAK2 and STAT3 mitigated RBP4‐induced myotube atrophy, indicating that RBP4 may promote muscle atrophy via STRA6/JAK2/STAT3 signalling. It is still worth noting that RBP4 also has pro‐inflammatory effects in endothelial cells,^34^ immune cells^6^, ^8^ and cardiomyocytes^14^, ^35^ through activating TLRs. Although we did not find any change in the expression of TLR2 and TLR4 between RBP4 knockout and wild‐type mice, additional in‐depth investigation is required to elucidate whether RBP4 induces skeletal muscle atrophy via its pro‐inflammatory effect and to identify other membrane receptors that may be responsible for the action of RBP4.

Our finding also provides potential clinical significance showing that lowering RBP4 by a specific pharmacological inhibitor A1120 leads to ameliorated fat infiltration and muscle atrophy in denervated mice. A1120 is a non‐retinoid RBP4 ligand that disrupts retinol‐induced RBP4‐transthyretin interaction, resulting in increased excretion of RBP4 through the kidneys.^36^ A1120 was originally developed for the improvement of insulin resistance^36^ and was later applied for the treatment of age‐related macular degeneration and inherited Stargardt macular dystrophy.^13^, ^37^ Unlike fenretinide, another RBP4 antagonist, A1120 is neither a retinoid nor an agonist to RARα, the property of which may avoid the retinoids‐associated side effects, such as nyctalopia and delayed dark adaptation.^37^ In addition, previous studies on different doses of A1120 did not observe any systemic toxicities in mice.^13^, ^36^, ^38^ Although there have been only research data in murine models, these preclinical results suggest that, if available, a safe RBP4 antagonist without any ocular side effect—like A1120—would be preferred for the treatment of degenerative muscle diseases.

In conclusion, our data reveal that RBP4 promotes fat infiltration and muscle atrophy through a STRA6‐dependent and JAK2/STAT3 pathway‐mediated mechanism in denervated skeletal muscle. Our findings not only shed light on the role of RBP4 in the progression of muscle atrophy but also provide a potential therapeutic approach by normalizing of RBP4 for the treatment of skeletal muscle degeneration.

## Conflict of interest statement

The authors declare no conflicts of interest.

## Supporting information


**Table S1.** **Sequences of siRNAs**.
**Table S2. Primer sets for Real‐time PCR analyses**.
**Table S3. Body weight and muscle mass in A1120‐treated mice**.
**Figure S1. Construction and genotyping of RBP4 knockout mice. (A)** The schematic diagram of construction of RBP4 knockout mice. **(B)** The primers (left) and representative images of genotyping. WT: wild type; KO: knockout.
**Figure S2. Denervation‐induced muscle atrophy in mice. (A)** Representative immunofluorescence staining of myofiber with Laminin (Red, upper) and myofiber cross‐sectional area of gastrocnemius (lower) in gastrocnemius. Scale bar = 50 μm. **(B)** Distribution of myofiber with different cross‐sectional area in gastrocnemius. **(C)** The mRNA levels of muscle atrophy marker Atrogin‐1 and MuRF1 as well as myogenic regulator MyoD and MyoG in gastrocnemius. *n* = 8 per group, Ctrl: control; DEN: denervation. For Supplementary Figure 2A, One‐way ANOVA analyses with post‐hoc correlation were used. For Supplementary Figure 2C, Student's *t* tests were used. *: *P <* 0.05; **: *P <* 0.01; NS: no significance.
**Figure S3. RBP4 expression is induced in denervated tibialis anterior muscles in mice.** Denervation‐induced muscle atrophy model was constructed in wild type mice. **(A‐B)** The mRNA **(A)** and protein **(B)** levels of RBP4 in tibialis anterior muscles. **(C)** Representative H&E staining of myofiber cross‐section of gastrocnemius (upper) and area of infiltrated fat in tibialis anterior muscles (lower). Arrow: infiltrated fat. Scale bar = 50 μm. **(D)** Immunofluorescence staining for RBP4 (Green) in infiltrated fatty region (Red) in tibialis anterior muscles. Scale bar = 100 μm. *n* = 8 per group, Ctrl: control; DEN: denervation. For Supplementary Figure 3A and 3B, Student's *t* tests were used. For Supplementary Figure 3C, One‐way ANOVA analyses with post‐hoc correlation were used. *: *P <* 0.05; **: *P <* 0.01; NS: no significance.
**Figure S4. Knockout of RBP4 attenuates denervation‐induced skeletal muscle atrophy in tibialis anterior muscles.** RBP4 knockout mice were applied and subjected to denervation procedure. **(A)** Immunofluorescence staining for RBP4 (Green) in the skeletal muscles. Scale bar = 100 μm. Gast: gastrocnemius; TA: tibialis anterior muscle. **(B)** Expression of genes related to muscle atrophy, myogenesis, muscle regeneration, and muscle fibre type transformation in tibialis anterior muscles. **(C)** Representative immunofluorescence staining of myofiber with Laminin (Red) (left) and myofiber cross‐sectional area (right) of tibialis anterior muscles. Scale bar = 50 μm. **(D)** Distribution of myofiber with different cross‐sectional area in tibialis anterior muscles. **(E)** The mRNA levels of muscle atrophy marker Atrogin‐1 and MuRF1, and myogenic regulator MyoD and MyoG in tibialis anterior muscles. (F) Representative H&E staining of myofiber cross‐section of tibialis anterior muscles (left) and area of infiltrated fat in tibialis anterior muscles (right). Arrow: infiltrated fat. Scale bar = 50 μm. *n* = 8 per group, Ctrl: control; DEN: denervation; WT: wild type; KO: knockout. Two‐way ANOVA analyses with post‐hoc correlation were used. *: *P <* 0.05; **: *P <* 0.01.
**Figure S5.** RBP4 aggregates denervation‐induced skeletal muscle atrophy tibialis anterior muscles. RBP4 knockout mice were subjected to denervation and then received the injection of either retinol‐free RBP4 (apo‐RBP4) or retinol‐bound RBP4 (holo‐RBP4) before sacrifice. (A) The mRNA levels of muscle atrophy marker Atrogin‐1 and MuRF1, and myogenic regulator MyoD and MyoG in tibialis anterior muscles from mice treated with apo‐RBP4. (B) The mRNA levels of muscle atrophy marker Atrogin‐1 and MuRF1, and myogenic regulator MyoD and MyoG in tibialis anterior muscles from mice treated with holo‐RBP4. (C) Representative immunofluorescence staining of myofiber with Laminin (Red, upper) and myofiber cross‐sectional area (lower) of tibialis anterior muscles from mice treated with apo‐RBP4 and holo‐RBP4. Scale bar = 50 μm. (D) Distribution of myofiber with different cross‐sectional area in tibialis anterior muscles from mice treated with apo‐RBP4 (upper) and holo‐RBP4 (lower). (E) Representative H&E staining of myofiber cross‐section of tibialis anterior muscles from mice treated with apo‐RBP4 and holo‐RBP4. Arrow: infiltrated fat. Scale bar = 50 μm. *n* = 8 per group, Ctrl: control; DEN: denervation. One‐way ANOVA analyses with post‐hoc correlation were used. *: *P <* 0.05; **: *P <* 0.01; NS: no significance.
**Figure S6.** (A) The change of the mRNA levels of myogenic regulator MyoG, MyoD, and MHC during the differentiation of C2C12 myotubes. (B) The mRNA levels of TLR2, TLR4, and STRA6 in gastrocnemius from wild type and RBP4 knockout mice. (C) The mRNA levels of TNF‐α, IL‐6, MCP‐1, and IL‐1β in gastrocnemius from wild type and RBP4 knockout mice. (D) The mRNA levels of TLR2, TLR4, and STRA6 in C2C12 myotubes treated with holo‐RBP4. (E) The protein level of STRA6 in C2C12 myotubes treated with siSTRA6. Ctrl: control; DEN: denervation; WT: wild type; KO: knockout. For Supplementary Figure 6B and 6C, Two‐way ANOVA analyses with post‐hoc correlation were used. For Supplementary 6D and 6E, One‐way ANOVA analyses with post‐hoc correlation were used. *: *P <* 0.05; **: *P <* 0.01; NS: no significance.
**Figure S7.** (A) Heatmap of the retinoid isomers. Red represents increase while blue represents decrease. (B‐E) The levels of retinol (B), retinal (C), retinyl ester (D), and retinoic acid (E) in the gastrointestinal muscle. (F) The expression of key genes involved in cellular retinol metabolism in the gastrointestinal muscle between RBP4 knockout and wild type mice. Comparison between two groups was performed with Mann–Whitney *U* tests. One‐way ANOVA analyses with post‐hoc correlation were used. *: *P <* 0.05; NS: no significance.
**Figure S8.** (A‐B) The mRNA levels of JAK1, JAK2, JAK3, and Tyk2 in gastrocnemius (A) and tibialis anterior muscles (B) from wild type and RBP4 knockout mice. (C‐D) The mRNA levels of STAT family in gastrocnemius (C) and tibialis anterior muscles (D) from wild type and RBP4 knockout mice. (E) The protein level of JAK2 in C2C12 myotubes treated with JAK antagonist AG490. (F) The protein level of STAT3 in C2C12 myotubes treated with siSTAT3. *n* = 8 per group for mice and n = 8 per group for C2C12 myotubes. Ctrl: control; DEN: denervation; WT: wild type; KO: knockout. For Supplementary Figure 8A‐8D, Two‐way ANOVA analyses with post‐hoc correlation were used. For Supplementary 8E and 8F, One‐way ANOVA analyses with post‐hoc correlation were used. *: *P <* 0.05; **: *P <* 0.01; NS: no significance.
**Figure S9.** The effect of A1120 on the gastrocnemius of sham hindlimbs. (A) The ratio of muscle weight to body weight. (B) Representative immunofluorescence staining of myofiber with Laminin (Red, upper) and myofiber cross‐sectional area of gastrocnemius from mice treated with A1120. Scale bar = 100 μm. (C) Distribution of myofiber with different cross‐sectional area in gastrocnemius from mice treated with A1120. (D) Representative H&E staining of myofiber cross‐section of gastrocnemius (upper) and area of infiltrated fat in gastrocnemius (lower) from mice treated with A1120. Scale bar = 50 μm. *n* = 8 per group. One‐way ANOVA analyses with post‐hoc correlation were used. NS: no significance.
**Figure S10.** Pharmacological RBP4 inhibitor alleviates denervation‐induced muscle atrophy in tibialis anterior muscles. (A) The ratio of muscle weight to body weight. (B) Representative immunofluorescence staining of myofiber with Laminin (Red, upper) and myofiber cross‐sectional area (lower) of tibialis anterior muscles from mice treated with A1120. Scale bar = 50 μm. (C) Distribution of myofiber with different cross‐sectional area in tibialis anterior muscles from mice treated with A1120. (D) Representative H&E staining of myofiber cross‐section of tibialis anterior muscles (upper) and area of infiltrated fat in tibialis anterior muscles (lower) from mice treated with A1120. Arrow: infiltrated fat. Scale bar = 50 μm. (E) The mRNA levels of muscle atrophy marker Atrogin‐1 and MuRF1 as well as myogenic regulator MyoD and MyoG in tibialis anterior muscles from mice treated with A1120. (F) The protein level of STRA6, JAK2, STAT3, muscle atrophy marker Atrogin‐1 and MuRF1, as well as myogenic regulator MyoD and MyoG in tibialis anterior muscles from mice treated with A1120. *n* = 8 per group, DEN: denervation. One‐way ANOVA analyses with post‐hoc correlation were used. *: *P <* 0.05; **: *P <* 0.01; NS: no significance.
**Figure S11.** The effect of A1120 on the tibialis anterior muscles of sham hindlimbs. (A) The ratio of muscle weight to body weight. (B) Representative immunofluorescence staining of myofiber with Laminin (Red, upper) and myofiber cross‐sectional area of tibialis anterior muscles from mice treated with A1120. Scale bar = 50 μm. (C) Distribution of myofiber with different cross‐sectional area in tibialis anterior muscles from mice treated with A1120. (D) Representative H&E staining of myofiber cross‐section of tibialis anterior muscles (upper) and area of infiltrated fat in tibialis anterior muscles (lower) from mice treated with A1120. Scale bar = 50 μm. *n* = 8 per group, DEN: denervation. One‐way ANOVA analyses with post‐hoc correlation were used. NS: no significance.

## Data Availability

The datasets used and/or analysed during the current study are available from the corresponding author on reasonable request.
