# Supplementary material for: RBP4 promotes denervation‐induced muscle atrophy through STRA6‐dependent pathway
Source: J Cachexia Sarcopenia Muscle. 2024 Jun 21;15(4):1601–15. doi: 10.1002/jcsm.13518 (PMC11294031; doi:10.1002/jcsm.13518)
Supplement: Supplementary file 1 — Table S1. Sequences of siRNAs. Table S2. Primer sets for Real‐time PCR analyses. Table S3. Body weight and muscle mass in A1120‐treated mice. Figure S1. Construction and genotyping of RBP4 knockout mice. (A) The schematic diagram of construction of RBP4 knockout mice. (B) The primers (left) and representative images of genotyping. WT: wild type; KO: knockout. Figure S2. Denervation‐induced muscle atrophy in mice. (A) Representative immunofluorescence staining of myofiber with Laminin (Red, upper) and myofiber cross‐sectional area of gastrocnemius (lower) in gastrocnemius. Scale bar = 50 μm. (B) Distribution of myofiber with different cross‐sectional area in gastrocnemius. (C) The mRNA levels of muscle atrophy marker Atrogin‐1 and MuRF1 as well as myogenic regulator MyoD and MyoG in gastrocnemius. n = 8 per group, Ctrl: control; DEN: denervation. For Supplementary Figure 2A, One‐way ANOVA analyses with post‐hoc correlation were used. For Supplementary Figure 2C, Student's t tests were used. *: P < 0.05; **: P < 0.01; NS: no significance. Figure S3. RBP4 expression is induced in denervated tibialis anterior muscles in mice. Denervation‐induced muscle atrophy model was constructed in wild type mice. (A‐B) The mRNA (A) and protein (B) levels of RBP4 in tibialis anterior muscles. (C) Representative H&E staining of myofiber cross‐section of gastrocnemius (upper) and area of infiltrated fat in tibialis anterior muscles (lower). Arrow: infiltrated fat. Scale bar = 50 μm. (D) Immunofluorescence staining for RBP4 (Green) in infiltrated fatty region (Red) in tibialis anterior muscles. Scale bar = 100 μm. n = 8 per group, Ctrl: control; DEN: denervation. For Supplementary Figure 3A and 3B, Student's t tests were used. For Supplementary Figure 3C, One‐way ANOVA analyses with post‐hoc correlation were used. *: P < 0.05; **: P < 0.01; NS: no significance. Figure S4. Knockout of RBP4 attenuates denervation‐induced skeletal muscle atrophy in tibialis anterior muscles. RBP4 knockout [file JCSM-15-1601-s001.docx]

**Supplementary Table 1. Sequences of siRNAs**

| Name | Sequence (5′ to 3′) |
| --- | --- |
| siSTRA6 | GAAGGCAGCCCTTACTAGT |
| siSTAT3 | GTGTGAGGTGCCTACTTGCTC |
| siControl | TTCTCCGAACGTGTCACGT |

**Supplementary Table 2. Primer sets for Real-time PCR analyses**

| Gene | Forward primer (5′ to 3′) | Reverse Primer (5′ to 3′) |
| --- | --- | --- |
| Atrogin-1 | CAGCTTCGTGAGCGACCTC | GGCAGTCGAGAAGTCCAGTC |
| MuRF1 | GTGTGAGGTGCCTACTTGCTC | GCTCAGTCTTCTGTCCTTGGA |
| Myog | GAGACATCCCCCTATTTCTACCA | GCTCAGTCCGCTCATAGCC |
| MyoD | CCACTCCGGGACATAGACTTG | AAAAGCGCAGGTCTGGTGAG |
| RBP4 | AGTCAAGGAGAACTTCGACAAGG | CAGAAAACTCAGCGATGATGTTG |
| C/EBPα | CAAGAACAGCAACGAGTACCG | GTCACTGGTCAACTCCAGCAC |
| PPARγ | TCGCTGATGCACTGCCTATG | GAGAGGTCCACAGAGCTGATT |
| Pax3 | AGTCAGATGAAGGCTCCG | CTCCTCCCTGGTGTAAATGTC |
| Pax7 | GCTCAGAATCAAGTTCGGG | CCCTCATCCAGACGGTT |
| Myh1 | CTCTTCCCGCTTTGGTAAGTT | CAGGAGCATTTCGATTAGATCCG |
| Myh2 | GCACCCATCCTCATTTCGTGA | GGAATGGCACTTGCGTTTAACA |
| Myh4 | CTTTGCTTACGTCAGTCAAGCT | AGCGCCTGTGAGCTTGTAAA |
| Myh7 | ACTGTCAACACTAAGAGGGTCA | TTGGATGATTTGATCTTCCAGGG |
| LRAT | CCGTCCCTATGAAATCAGCTC | ATGGGCGACACGGTTTTCC |
| CRABP1 | CAGCAGCGAGAATTTCGACGA | CGCACAGTAGTGGATGTCTTGA |
| CRABP2 | ATGCCTAACTTTTCTGGCAACT | GCACAGTGGTGGAGGTTTTGA |
| RDH1 | GTCATGGGCCGAATGTCTTTC | CACAAGTCTTGAAGCCTCCAG |
| RDH10 | GAACATCGTAGTGGAGTTCTTCG | CGGTCTCCTCATTGCTCTGC |
| ALDH1a1 | GTGAAAAGGAGTGTTGAGCGA | TTTGTTCCCCCAGCGTC |
| ALDH1a2 | CAGAGAGTGGGAGAGTGTTCC | CACACAGAACCAAGAGAGAAGG |
| ALDH1a3 | GGGTCACACTGGAGCTAGGA | CTGGCCTCTTCTTGGCGAA |
| RARα | CCCTGAACCGGACTCAGAT | AGGTCCTGTCGGTCCTCCAC |
| RARβ | TGCTTTGAAGTGGGCATGT | TTACCCAGCTGGCAGAGTG |
| RARγ | GACCCAGCCAACCCTACAT | ACATCTCCGGGTTCTCCAG |
| RXRα | ATGAGAACGAGGTGGAGT | TGCTGCTTGACAGATGTTGGTAA |
| RXRβ | GAAGAGTGACCAAGGCG | GTAGGGAGGAGAAGTGCG |
| RXRγ | ATCTACACCTGTCGGGATAAC | TGGCTACTACTGGCACATTC |
| TLR2 | GCAAACGCTGTTCTGCTCAG | AGGCGTCTCCCTCTATTGTATT |
| TLR4 | ATGGCATGGCTTACACCACC | GAGGCCAATTTTGTCTCCACA |
| STRA6 | CTGGTACATCGAGGAACCTCT | CCAGGAACGACAGTGAAGCC |
| JAK1 | CTCTCTGTCACAACCTCTTCGC | TTGGTAAAGTAGAACCTCATGCG |
| JAK2 | TTGTGGTATTACGCCTGTGTATC | ATGCCTGGTTGACTCGTCTAT |
| JAK3 | GGCGTGGCGGTTAGTAAAGAA | CCCCCTATCTAGTCTCACCCT |
| Tyk2 | AGCCATCTTGGAAGACAGCAA | GACTTTGTGTGCGATGTGGAT |
| STAT1 | TCACAGTGGTTCGAGCTTCAG | GCAAACGAGACATCATAGGCA |
| STAT2 | TCCTGCCAATGGACGTTCG | GTCCCACTGGTTCAGTTGGT |
| STAT3 | CAATACCATTGACCTGCCGAT | GAGCGACTCAAACTGCCCT |
| STAT4 | TGGCAACAATTCTGCTTCAAAAC | GAGGTCCCTGGATAGGCATGT |
| STAT5a | CGCCAGATGCAAGTGTTGTAT | TCCTGGGGATTATCCAAGTCAAT |
| STAT5b | CGATGCCCTTCACCAGATG | AGCTGGGTGGCCTTAATGTTC |
| STAT6 | CTCTGTGGGGCCTAATTTCCA | CATCTGAACCGACCAGGAACT |
| IL-1β | ATGAGAGCATCCAGCTTCAA | TGAAGGAAAAGAAGGTGCTC |
| IL-6 | GAGGATACCACTCCCAACAGACC | AAGTGCATCATCGTTGTTCATACA |
| TNF-α | ACCCTGGTATGAGCCCATATAC | ACACCCATTCCCTTCACAGAG |
| MCP-1 | GCATCTGCCCTAAGGTCTTCA | TGCTTGAGGTGGTTGTGGAA |
| GAPDH | AGGTCGGTGTGAACGGATTTG | TGTAGACCATGTAGTTGAGGTCA |

**Supplementary Table 3. Body weight and muscle mass in A1120-treated mice**

|  | Control | DMSO | 10 mg/kg | 30 mg/kg | 50 mg/kg |
| --- | --- | --- | --- | --- | --- |
| Body weight | 17.76±0.56 | 17.75±0.78 | 17.80±0.67 | 17.79±0.45 | 17.77±0.75 |
| Sham gastrocnemius | 139.42±15.75 | 138.72±13.89 | 137.62±11.23 | 138.15±13.54 | 139.65±12.37 |
| Injured gastrocnemius | 138.45±13.25 | 136.40±13.68 | 114.15±7.54 | 96.65±7.37 | 77.05±13.65 |
| Sham tibialis anterior muscle | 13.82±1.75 | 13.28±1.67 | 13.56±1.37 | 13.89±3.26 | 13.79±3.89 |
| Injured tibialis anterior muscle | 13.92±1.87 | 13.45±2.78 | 11.76±2.45 | 9.82±1.34 | 7.82±0.89 |

Data are presented as mean±SD.

**Supplementary Figure legends**

**Supplementary Figure 1. Construction and genotyping of RBP4 knockout mice. (A)** The schematic diagram of construction of RBP4 knockout mice. **(B)** The primers (left) and representative images of genotyping. WT: wild type; KO: knockout.

**Supplementary Figure 2. Denervation-induced muscle atrophy in mice. (A)** Representative immunofluorescence staining of myofiber with Laminin (Red, upper) and myofiber cross-sectional area of gastrocnemius (lower) in gastrocnemius. Scale bar =50 μm. **(B)** Distribution of myofiber with different cross-sectional area in gastrocnemius. **(C)** The mRNA levels of muscle atrophy marker Atrogin-1 and MuRF1 as well as myogenic regulator MyoD and MyoG in gastrocnemius. n=8 per group, Ctrl: control; DEN: denervation. For Supplementary Figure 2A, One-way ANOVA analyses with post-hoc correlation were used. For Supplementary Figure 2C, Student’s *t* tests were used. *: *P<*0.05; **: *P<*0.01; NS: no significance.

**Supplementary Figure 3. RBP4 expression is induced in denervated tibialis anterior muscles in mice.** Denervation-induced muscle atrophy model was constructed in wild type mice. **(A-B)** The mRNA **(A)** and protein **(B)** levels of RBP4 in tibialis anterior muscles. **(C)** Representative H&E staining of myofiber cross-section of gastrocnemius (upper) and area of infiltrated fat in tibialis anterior muscles (lower). Arrow: infiltrated fat. Scale bar =50 μm. **(D)** Immunofluorescence staining for RBP4 (Green) in infiltrated fatty region (Red) in tibialis anterior muscles. Scale bar = 100 μm. n=8 per group, Ctrl: control; DEN: denervation. For Supplementary Figure 3A and 3B, Student’s *t* tests were used. For Supplementary Figure 3C, One-way ANOVA analyses with post-hoc correlation were used. *: *P<*0.05; **: *P<*0.01; NS: no significance.

**Supplementary Figure 4. Knockout of RBP4 attenuates denervation-induced skeletal muscle atrophy in tibialis anterior muscles.** RBP4 knockout mice were applied and subjected to denervation procedure. **(A)** Immunofluorescence staining for RBP4 (Green) in the skeletal muscles. Scale bar = 100 μm. Gast: gastrocnemius; TA: tibialis anterior muscle. **(B)** Expression of genes related to muscle atrophy, myogenesis, muscle regeneration, and muscle fiber type transformation in tibialis anterior muscles. **(C)** Representative immunofluorescence staining of myofiber with Laminin (Red) (left) and myofiber cross-sectional area (right) of tibialis anterior muscles. Scale bar =50 μm. **(D)** Distribution of myofiber with different cross-sectional area in tibialis anterior muscles. **(E)** The mRNA levels of muscle atrophy marker Atrogin-1 and MuRF1, and myogenic regulator MyoD and MyoG in tibialis anterior muscles. **(F)** Representative H&E staining of myofiber cross-section of tibialis anterior muscles (left) and area of infiltrated fat in tibialis anterior muscles (right). Arrow: infiltrated fat. Scale bar =50 μm. n=8 per group, Ctrl: control; DEN: denervation; WT: wild type; KO: knockout. Two-way ANOVA analyses with post-hoc correlation were used. *: *P<*0.05; **: *P<*0.01.

**Supplementary Figure 5. RBP4 aggregates denervation-induced skeletal muscle atrophy tibialis anterior muscles.** RBP4 knockout mice were subjected to denervation and then received the injection of either retinol-free RBP4 (apo-RBP4) or retinol-bound RBP4 (holo-RBP4) before sacrifice. **(A)** The mRNA levels of muscle atrophy marker Atrogin-1 and MuRF1, and myogenic regulator MyoD and MyoG in tibialis anterior muscles from mice treated with apo-RBP4. **(B)** The mRNA levels of muscle atrophy marker Atrogin-1 and MuRF1, and myogenic regulator MyoD and MyoG in tibialis anterior muscles from mice treated with holo-RBP4. **(C)** Representative immunofluorescence staining of myofiber with Laminin (Red, upper) and myofiber cross-sectional area (lower) of tibialis anterior muscles from mice treated with apo-RBP4 and holo-RBP4. Scale bar =50 μm. **(D)** Distribution of myofiber with different cross-sectional area in tibialis anterior muscles from mice treated with apo-RBP4 (upper) and holo-RBP4 (lower). **(E)** Representative H&E staining of myofiber cross-section of tibialis anterior muscles from mice treated with apo-RBP4 and holo-RBP4. Arrow: infiltrated fat. Scale bar =50 μm. n=8 per group, Ctrl: control; DEN: denervation. One-way ANOVA analyses with post-hoc correlation were used. *: *P<*0.05; **: *P<*0.01; NS: no significance.

**Supplementary Figure 6. (A)** The change of the mRNA levels of myogenic regulator MyoG, MyoD, and MHC during the differentiation of C2C12 myotubes. **(B)** The mRNA levels of TLR2, TLR4, and STRA6 in gastrocnemius from wild type and RBP4 knockout mice. **(C)** The mRNA levels of TNF-α, IL-6, MCP-1, and IL-1β in gastrocnemius from wild type and RBP4 knockout mice. **(D)** The mRNA levels of TLR2, TLR4, and STRA6 in C2C12 myotubes treated with holo-RBP4. **(E)** The protein level of STRA6 in C2C12 myotubes treated with siSTRA6. Ctrl: control; DEN: denervation; WT: wild type; KO: knockout. For Supplementary Figure 6B and 6C, Two-way ANOVA analyses with post-hoc correlation were used. For Supplementary 6D and 6E, One-way ANOVA analyses with post-hoc correlation were used. *: *P<*0.05; **: *P<*0.01; NS: no significance.

**Supplementary Figure 7.** **(A)** Heatmap of the retinoid isomers. Red represents increase while blue represents decrease. **(B-E)** The levels of retinol **(B)**, retinal **(C)**, retinyl ester **(D)**, and retinoic acid **(E)** in the gastrointestinal muscle. **(F)** The expression of key genes involved in cellular retinol metabolism in the gastrointestinal muscle between RBP4 knockout and wild type mice. Comparison between two groups was performed with Mann-Whitney *U* tests. One-way ANOVA analyses with post-hoc correlation were used. *: *P<*0.05; NS: no significance.

**Supplementary Figure 8. (A-B)** The mRNA levels of JAK1, JAK2, JAK3, and Tyk2 in gastrocnemius **(A)** and tibialis anterior muscles **(B)** from wild type and RBP4 knockout mice. **(C-D)** The mRNA levels of STAT family in gastrocnemius **(C)** and tibialis anterior muscles **(D)** from wild type and RBP4 knockout mice. **(E)** The protein level of JAK2 in C2C12 myotubes treated with JAK antagonist AG490. **(F)** The protein level of STAT3 in C2C12 myotubes treated with siSTAT3. n=8 per group for mice and n=8 per group for C2C12 myotubes. Ctrl: control; DEN: denervation; WT: wild type; KO: knockout. For Supplementary Figure 8A-8D, Two-way ANOVA analyses with post-hoc correlation were used. For Supplementary 8E and 8F, One-way ANOVA analyses with post-hoc correlation were used. *: *P<*0.05; **: *P<*0.01; NS: no significance.

**Supplementary Figure 9. The effect of A1120 on the gastrocnemius of sham hindlimbs. (A)** The ratio of muscle weight to body weight. **(B)** Representative immunofluorescence staining of myofiber with Laminin (Red, upper) and myofiber cross-sectional area of gastrocnemius from mice treated with A1120. Scale bar =100 μm. **(C)** Distribution of myofiber with different cross-sectional area in gastrocnemius from mice treated with A1120. **(D)** Representative H&E staining of myofiber cross-section of gastrocnemius (upper) and area of infiltrated fat in gastrocnemius (lower) from mice treated with A1120. Scale bar =50 μm. n=8 per group. One-way ANOVA analyses with post-hoc correlation were used. NS: no significance.

**Supplementary Figure 10. Pharmacological RBP4 inhibitor alleviates denervation-induced muscle atrophy in tibialis anterior muscles. (A)** The ratio of muscle weight to body weight. **(B)** Representative immunofluorescence staining of myofiber with Laminin (Red, upper) and myofiber cross-sectional area (lower) of tibialis anterior muscles from mice treated with A1120. Scale bar =50 μm. **(C)** Distribution of myofiber with different cross-sectional area in tibialis anterior muscles from mice treated with A1120. **(D)** Representative H&E staining of myofiber cross-section of tibialis anterior muscles (upper) and area of infiltrated fat in tibialis anterior muscles (lower) from mice treated with A1120. Arrow: infiltrated fat. Scale bar =50 μm. **(E)** The mRNA levels of muscle atrophy marker Atrogin-1 and MuRF1 as well as myogenic regulator MyoD and MyoG in tibialis anterior muscles from mice treated with A1120. **(F)** The protein level of STRA6, JAK2, STAT3, muscle atrophy marker Atrogin-1 and MuRF1, as well as myogenic regulator MyoD and MyoG in tibialis anterior muscles from mice treated with A1120. n=8 per group, DEN: denervation. One-way ANOVA analyses with post-hoc correlation were used. *: *P<*0.05; **: *P<*0.01; NS: no significance.

**Supplementary Figure 11. The effect of A1120 on the tibialis anterior muscles of sham hindlimbs. (A)** The ratio of muscle weight to body weight. **(B)** Representative immunofluorescence staining of myofiber with Laminin (Red, upper) and myofiber cross-sectional area of tibialis anterior muscles from mice treated with A1120. Scale bar =50 μm. **(C)** Distribution of myofiber with different cross-sectional area in tibialis anterior muscles from mice treated with A1120. **(D)** Representative H&E staining of myofiber cross-section of tibialis anterior muscles (upper) and area of infiltrated fat in tibialis anterior muscles (lower) from mice treated with A1120. Scale bar =50 μm. n=8 per group, DEN: denervation. One-way ANOVA analyses with post-hoc correlation were used. NS: no significance.
